# Genetic and phenotypic variation of the malaria vector *Anopheles atroparvus *in southern Europe

**DOI:** 10.1186/1475-2875-10-5

**Published:** 2011-01-11

**Authors:** José L Vicente, Carla A Sousa, Bulent Alten, Selim S Caglar, Elena Falcutá, José M Latorre, Celine Toty, Hélène Barré, Berna Demirci, Marco Di Luca, Luciano Toma, Ricardo Alves, Patrícia Salgueiro, Teresa L Silva, Maria D Bargues, Santiago Mas-Coma, Daniela Boccolini, Roberto Romi, Gabriela Nicolescu, Virgílio E do Rosário, Nurdan Ozer, Didier Fontenille, João Pinto

**Affiliations:** 1 Centro de Malária e outras Doenças Tropicais/UEI Malária, Instituto de Higiene e Medicina Tropical, Universidade Nova de Lisboa. Rua da Junqueira 100, 1349-008 Lisbon, Portugal; 2 UEI Entomologia Médica, Instituto de Higiene e Medicina Tropical, Universidade Nova de Lisboa, Lisbon, Portugal; 3 Faculty of Science, Hacettepe University, Ankara, Turkey; 4 National Institute for Research and Development in Microbiology and Immunology "Cantacuzino", Bucharest, Romania; 5 Departament de Parasitologia, Facultat de Farmàcia, Universitat de València, Valencia, Spain; 6 Intitut de Recherche pour le Développement, Montpellier, France; 7 CNRS UMR 6134, Laboratoire Parasites et Ecosystèmes Méditerranéens, Université de Corse, Corte, France; 8 Istituto Supperiore di Sanità, Rome, Italy

## Abstract

**Background:**

There is a growing concern that global climate change will affect the potential for pathogen transmission by insect species that are vectors of human diseases. One of these species is the former European malaria vector, *Anopheles atroparvus*. Levels of population differentiation of *An. atroparvus *from southern Europe were characterized as a first attempt to elucidate patterns of population structure of this former malaria vector. Results are discussed in light of a hypothetical situation of re-establishment of malaria transmission.

**Methods:**

Genetic and phenotypic variation was analysed in nine mosquito samples collected from five European countries, using eight microsatellite loci and geometric morphometrics on 21 wing landmarks.

**Results:**

Levels of genetic diversity were comparable to those reported for tropical malaria vectors. Low levels of genetic (0.004 <*F*_*ST *_<0.086) and phenotypic differentiation were detected among *An. atroparvus *populations spanning over 3,000 km distance. Genetic differentiation (0.202 <*F*_*ST *_<0.299) was higher between the sibling species *An. atroparvus *and *Anopheles maculipennis *s.s. Differentiation between sibling species was not so evident at the phenotype level.

**Conclusions:**

Levels of population differentiation within *An. atroparvus *were low and not correlated with geographic distance or with putative physical barriers to gene flow (Alps and Pyrenées). While these results may suggest considerable levels of gene flow, other explanations such as the effect of historical population perturbations can also be hypothesized.

## Background

Under the present scenario of human-driven environmental changes, global climate change is one the most relevant concerns. Climatic predictions point to a significant increase of summer droughts in south-western European regions over the next 60 years, but there is also an increased risk for more frequent flash floods during the same period [1]. Since the life cycles and distribution of many insect vector species are directly influenced by climatological conditions, climate change has the potential to affect the incidence, seasonal transmission and geographic range of several vector-borne diseases [2]. It is still not clear, however, if the impact of climate change will be beneficial or adverse. Mosquito populations may tend to expand with warming and changes in rainfall patterns, which will tend to increase disease transmission. On the other hand, mosquito reproduction and survival could be impaired by altered rainfall and increased aridity leading to a reduction in transmission [2]. Nonetheless, the overall effect of anthropogenic climate change on vector-borne diseases remains debated, and the outcome may vary regionally [3].

Malaria is the vector-borne disease with the highest impact in the World's human population. In 2008, there were *ca. *243 million cases, and an estimated 863,000 deaths attributed to malaria [4]. Although at present malaria endemic areas are mainly restricted to tropical and subtropical regions, several models project a geographical expansion of potential malaria transmission in the next few decades, and more substantial changes later this century [2].

Malaria was endemic in Europe until the mid 20^th ^century [5]. The eradication of malaria in the European region was largely due to a combination of changes in farming and husbandry, improvement in house construction and vector control. However, in recent years, the disease re-emerged in residual foci in Eastern Europe (Azerbaijan, Georgia, Kyrgyzstan, Tajikistan, Turkey and Uzbekistan), resulting in more than 30,000 malaria cases in the year 2000 [4]. Since then, intensive control activities have been re-implemented throughout the affected region, and the number of reported cases has been reduced substantially to 660 in 2008 [4].

Although the risk of malaria re-emergence is uncertain for Western/Southern European countries, the present climate change situation gave rise to some concern. One of the reasons was a predicted increase in mosquito vectorial capacity, especially in the southern countries of Europe and the Mediterranean [6]. This in conjunction with the increasing intercontinental human movement may favour the re-establishment of autochthonous malaria transmission.

The former European malaria vectors were mainly members of the *Anopheles maculipennis *complex that are still widely distributed throughout the continent [7]. This complex comprises 13 Palearctic sibling species, of which *Anopheles atroparvus*, *Anopheles labranchiae *and *Anopheles sacharovi *were the main malaria vectors in the European region. In Europe, the distribution of *An. atroparvus *ranges from Britain to Russia (north Caucasus). It is absent in some Mediterranean regions, such as southern Italy, Greece and Turkey [8].

Because its importance as a disease vector has declined, research on the biology of *An*. *atroparvus *and its sibling species has decreased in the last decades. However, the concern with malaria re-emergence has resulted in a revival of interest in European *Anopheles *mosquitoes. In this context, knowledge about the population structure and levels of gene flow in this species is of major importance to infer the potential for the re-establishment and spreading of malaria transmission under a scenario of local introduction of parasites. Furthermore, if necessary, it will be a critical tool for the design of vector control plans. In this study, genetic and phenotypic variation was analysed, using microsatellites and geometric morphometrics, in order to determine patterns of population structure of *An. atroparvus *in Southern Europe.

## Methods

### Mosquito collections

**Figure 1:**
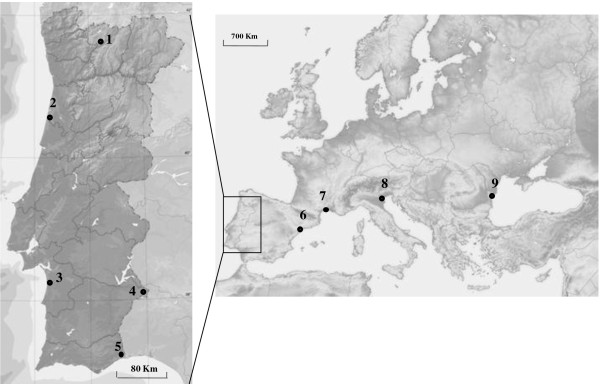
**Maps of Europe and Portugal showing the location of collection sites**. 1: Montalegre (sample collected in 2008), 2: Aveiro (2008), 3: Comporta (2007), 4: Barrancos (2008), 5: Castro Marim (2008), 6: Tarragona (2008), 7: Mèjanes (2008), 8: Venice (2006), 9: Salcioara (2008)

Adult mosquitoes were sampled by indoors resting collections (usually from animal shelters) that took place in summer time (June to October) between 2006 and 2008 in 9 collection sites from five European countries (Figure 1). All sites were rural. The localities in Romania, Italy, France, Spain and three sites in Portugal (2, 3 and 5, Figure 1) are located in coastal areas characterized by wetlands with the presence of rice fields. Sites 1 and 4 (Figure 1) are located, respectively, in drier mountainous and plain inland regions of Portugal. Mosquitoes were identified to species or species complex by stereomicroscopic observation of morphologic characters using identification keys [9,10]. All the specimens were preserved individually at 4°C or room temperature in tubes filled with silica gel, until further analysis.

### Molecular identification of sibling species

Genomic DNA was extracted from single mosquitoes following a phenol-chloroform procedure [11]. Species identification of four members of the *An. maculipennis *complex was carried out by PCR-RFLP using protocols derived from those described by Proft *et al *[12]. The Internal Transcribed Spacer 2 (ITS2) of the ribosomal DNA was amplified using primers for the conserved regions 5.8S and 28S [13]. PCR was carried out in a 25 μl volume containing 1X GoTaq^® ^Flexi Buffer (Promega, USA), 2.5 mM MgCl_2_, 200 μM dNTP's (Promega, USA), 5 μM of each primer and 1U of Go Taq polymerase (Promega, USA). The thermal cycling profile was as follows: 94°C for 5 min; 34 cycles of 94°C for 30 sec, 53°C for 30 sec and 72°C for 30 sec, followed by a final extension at 72°C for 7 min. A RFLP protocol allowed the identification of four sibling species of the *An. maculipennis *complex (*An. atroparvus*, *Anopheles labranchiae, Anopheles maculipennis *s.s. and *Anopheles melanoon*). For the restriction reaction, 5 μl of each ITS2 PCR product was added to 1X restriction enzyme buffer (buffer L, Roche Diagnostics, Germany) and 1.25U of *Cfo 1 *(Roche Diagnostics, Germany) enzyme, in a total volume of 20 μl, followed by incubation for 3 h at 37°C. Digested fragments were separated by electrophoresis on an ethidium bromide stained 2% agarose gel, showing sizes that were diagnostic for each species. For those specimens presenting restriction fragments with lengths compatible with *An. labranchiae/An. maculipennis *s.s. (300 bp), a second enzymatic digestion was performed with *Hpa*II (Roche Diagnostics, Germany) using the same RFLP protocol as above. The diagnostic restriction patterns are: *An. atroparvus *(389 bp fragment), *An. melanoon *(fragments with 108 bp and 135 bp) and after the second enzymatic digestion *An. labranchiae *(279 bp fragment) and *An. maculipennis *s.s. (201 bp fragment).

### Microsatellite genotyping

Ten dinucleotide repeat microsatellites were amplified using fluorescently labelled (6-FAM, NED, or HEX; Applied Biosystems, USA) primers previously described [14]. PCR reactions were conducted in a 20 μl PCR mix containing 1x PCR GoTaq^® ^Flexi Buffer (Promega) 1.5 mM MgCl_2_, 200 μM dNTPs, 0.5 μM of each primer, 0.5U of Taq polymerase and 1 μl of DNA template. Cycling conditions included an initial denaturation at 94°C for 5 min, followed by 30 cycles of 94°C for 30 sec, annealing temperature (Ta: 50°C for MacuW161; 52°C for MacuGQ, MacuW149, MacuI3, MacuU182, MacuG66; 54°C for MacuUF, MacuQ72, MacuO177, MacuO185) for 30 sec, elongation at 72°C for 30 sec; and a final extension step of 5 min at 72°C. Amplified fragments were separated by capillary electrophoresis in an automatic sequencer (ABI 3730 Applied Biosystems, USA) at Yale's DNA Analysis Facility at Science Hill. Fragment sizes were scored using GeneMarker v1.4 (SoftGenetics, USA).

### Genetic data analysis

Allele diversity (*i.e. *number of different alleles, *A*), expected heterozygosity (*H*_*e*_) inbreeding coefficient (*F*_IS_) and tests for differences of *A *and *H*_*e *_among groups were computed using FSTAT v. 2.9.3.2 [15]. Tests of departures from Hardy-Weinberg proportions and linkage disequilibrium between pairs of loci were performed using the randomization approach implemented in FSTAT. The software MICRO-CHECKER [16] was used to search for null alleles at loci/samples. Based on these results, a null alleles corrected dataset was obtained following the procedure of Chapuis and Estoup [17] implemented by the software FREENA. This corrected dataset was used in the subsequent analysis of genetic differentiation.

A permutation test available in the software SPAGEDI v. 1.3a [18] was used (with the uncorrected database) to decide about the most appropriate mutation model for the microsatellite data set (*i.e. *infinite alleles model-IAM or stepwise-mutation model-SMM) and hence which differentiation statistics would better describe the genetic structure of the populations sampled. Briefly, allele size at each locus was randomly permuted among allelic states (20,000 permutations) to simulate a distribution of expected *R*_ST _values (p*R*_ST_) and 95% confidence intervals (CI) under the null hypothesis that differences in allele sizes do not contribute to population differentiation [19].

Genetic differentiation among samples was quantified by pairwise *F*_ST _estimates calculated according to Weir and Cockerham [20] using ARLEQUIN v. 3.11 [21]. With the aim of testing isolation by distance, pairwise estimates of Slatkin's linearized *F*_*ST *_[22] were tested for correlation with logarithmic (ln) geographic distances through Mantel tests available in ARLEQUIN. Factorial correspondence analysis over populations was performed based on pairwise allelic differences using GENETIX v4.03 [23]. This method allows to graphically representing multilocus genetic distances in two- or three dimensions so that the relationships between populations are determined by the way individuals cluster in the dimension plot.

Sequential Bonferroni corrections were used to adjust critical probability values for multiple tests in order to minimize type I errors [24].

### Geometric morphometric analysis

Morphometric analysis was performed with four samples collected in Portugal (Castro Marim, *N *= 45; Aveiro, *N *= 52; Barrancos, *N *= 84; Montalegre, *N *= 61) and the samples from Tarragona/Spain (*N *= 66), Mèjanes/France (*N *= 47), Venice/Italy (*N *= 44) and Salcioara/Romania (*N *= 29) (Figure 1). All specimens were screened for the presence of ecto- and endoparasites to prevent possible traumatic variations affecting the morphometric data. Before molecular analysis, the wings were detached from each specimen with forceps and stained for observation of veins using the following procedure: the wings were kept in 5% KOH solution for 20 min to remove scales. The wings were then placed in 95% ethanol for <10 sec, after which they were transferred to cups containing distilled water for washing. After staining, wings were mounted on labelled slides and coverslips with Entellan^® ^(Merck, Germany) medium. Slides were photographed using a Leica^® ^MZ-7.5 stereoscopic zoom dissection microscope with a DC-300 digital camera system. In order to reduce the measurement error, specimens were digitalized twice and scored by the same person. The second session of measurement was conducted after specimens were removed and replaced under the stereomicroscope in order to take positioning error into account [25].

**Figure 2:**
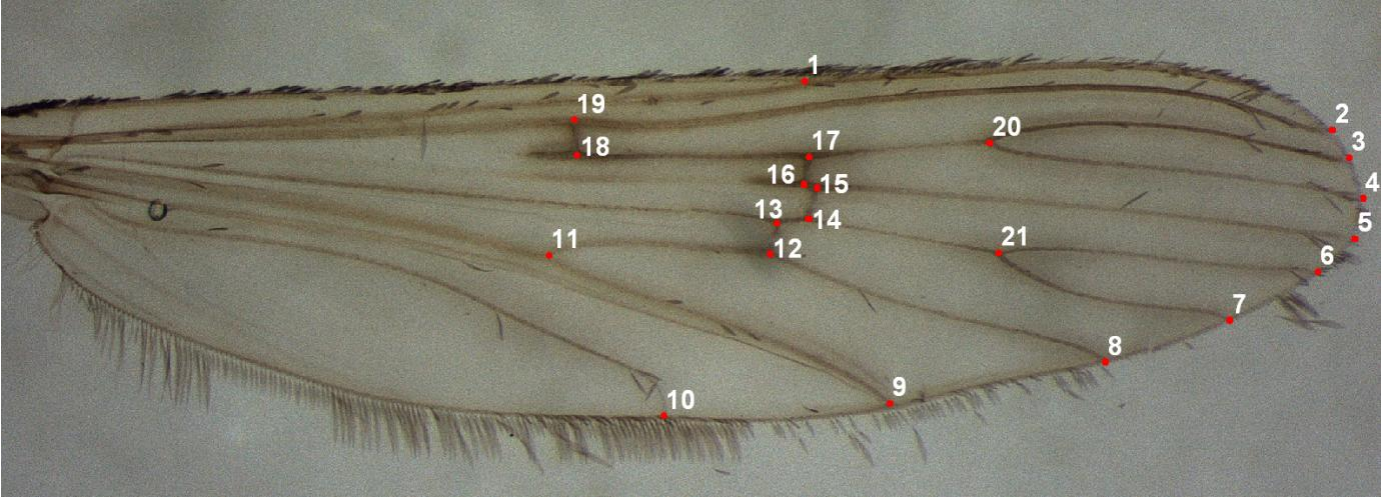
**Location of the 21 wing landmarks used in the morphometric analysis of *Anopheles maculipennis *s.l**.

Twenty-one landmarks of the left wings of mosquitoes were used for the analysis, following the methods described in Rohlf [26] and Slice [27] (Figure 2). The landmark configurations were scaled, translated and rotated against the consensus configuration by Generalized Procrustes Analysis (GPA, formerly termed GLS) [28,29] and used in Morphologika^® ^[30] to calculate centroid sizes and to perform principal components analysis (PCA). The size morphometry of samples was investigated by using the centroid sizes of the wings as an estimator. Centroid size is the square root of the sum of squared distances of a set of landmarks from their centroid [28]. The non-parametric Kruskal-Wallis test was used to determine differences in centroid sizes between samples.

A correlation matrix between samples was constructed using squared Mahalanobis distances (*D*_*M*_), available in the software STATISTICA 9.0 (StatSoft, USA). The Mahalanobis distance takes into account the covariance among the variables in calculating distances and it is widely used in cluster analysis and other classification techniques. With this measure, the problems of scale and correlation inherent to Euclidean distances are no longer an issue.

## Results

### Species identification

A total of 473 female mosquitoes of the *An. maculipennis *complex were analysed in this study. Of these, the specimens collected in Montalegre (*N *= 61), Portugal, were all identified as *An. maculipennis *s.s., while the remaining samples were identified as *An. atroparvus*.

### Genetic variability

Samples of 45 individuals from each collection site were used for microsatellite analysis. Of the 10 microsatellites analysed, two (MacuO185 and MacuW149) were monomorphic in most populations, and were thus excluded from further analysis. The mean expected heterozygosity per locus in *An. atroparvus *varied between 0.588 (MacuUF) and 0.876 (MacuI3) (see Additional file 1). Comporta (Portugal) showed the highest genetic variability (*H*_*e *_= 0.734; *A *= 8) while the sample from Romania had the lowest (*H*_*e *_= 0.608; *A *= 6). Samples from Portugal and France showed significantly higher values of *A *and *H*_*e *_(*P *< 0.03; 10,000 permutations) than the samples from Spain, Italy and Romania. The only sample of *An. maculipennis *s.s. revealed a smaller *H*_*e *_(0.504) and *A *(5) than the least diverse sample of *An. atroparvus *(Romania).

Exact tests of linkage disequilibrium between pairs of loci were non-significant for all the samples after Bonferroni corrections (*P *> 0.0027; adjusted significance level for 28 pair-wise tests per sample: α' = 0.0018).

There were 15 significant single-locus tests of Hardy-Weinberg proportions out of 72 performed (see Additional file 1). These were associated with high positive *F*_*IS *_values, indicating heterozygote deficits and were mostly concentrated at locus MacuG66 (3 out of 9 tests), MacuO177 (5 out of 9 tests) and MacuGQ (4 out of 9 tests). Coincidently, these loci exhibited the presence of null alleles in most samples analysed as revealed by MICROCHECKER (MacuG66 and MacuO177: 7/9 samples; MacuGQ: 5/9 samples). For these loci, a corrected genotypic database was obtained according to the procedures described in [17] and implemented in FREENA. This database was used in the subsequent analysis of genetic differentiation.

### Genetic differentiation

Single-locus permutation tests performed with the uncorrected database to assess the effect of stepwise mutations on population differentiation [19] were marginally significant at three out of the eight loci analysed (0.007≤ *P *≤ 0.028, see Additional file 1). When sequential Bonferroni corrections were applied these tests were found non-significant (corrected α'= 0.006, 8 tests). These results suggest only a weak effect of stepwise mutations compared to that of genetic drift in shaping genetic differentiation among samples. In this situation, allele identity based statistics such as *F*_*ST *_should be preferred over allele size based statistics [19].

Pairwise *F*_*ST *_estimates between samples are shown in Table 1. Within *An. atroparvus*, there were seven non-significant *F*_*ST *_estimates. Of these, one involved samples from Portugal and France while the others were all between Portuguese samples. In comparisons between countries, the highest *F*_*ST *_values involved the sample from Romania (0.052 ≤ *F*_*ST *_≤ 0.086) whereas the lowest were detected between Portugal and France (0.010 ≤ *F*_*ST *_≤ 0.016). There was a significant correlation between linearized *F*_*ST *_and geographic distance among *An. atroparvus *samples (Mantel Test: *P *= 0.022). However, when Romania was removed from the analysis, the correlation was no longer significant (Mantel Test: *P *= 0.181).

**Table 1:** Pairwise estimates of *F*_*ST *_(below diagonal) and *D*_*M *_(above diagonal) among populations of *Anopheles atroparvus*

	C. Marim	Barrancos	Comporta	Aveiro	Tarragona	Mèjanes	Venice	Salcioara	*macu*
C. Marim	-	8.8	n.d.	421.3	43.0	106.8	177.9	539.4	285.2
Barrancos	0.004	-	n.d.	311.7	13.8	56.1	109.7	414.4	196.6
Comporta	0.012	0.011	-	n.d.	n.d.	n.d.	n.d.	n.d.	n.d.
Aveiro	0.007	0.001	0.012	-	197.4	105.4	54.6	11.9	15.3
Tarragona	**0.054**	**0.057**	**0.048**	**0.066**	-	15.5	47.3	281.1	108.6
Mèjanes	**0.016**	**0.016**	0.010	**0.014**	**0.066**	-	10.4	167.8	44.4
Venice	**0.033**	**0.031**	**0.022**	**0.035**	**0.058**	**0.026**	-	102.1	14.8
Salcioara	**0.075**	**0.080**	**0.064**	**0.086**	**0.085**	**0.052**	**0.069**	-	43.9

*macu*	**0.221**	**0.228**	**0.202**	**0.220**	**0.245**	**0.230**	**0.263**	**0.299**	-

Above diagonal: *D*_*M *_based on morphometric measurements. n.d.: not-done, morphometric data was not available for the sample from Comporta. Below diagonal: estimates of *F*_*ST *_(significant values after Bonferroni corrections are in bold) based on microsatellites data. *macu*: *An. maculipennis *s.s. from Montalegre, Portugal.

**Figure 3:**
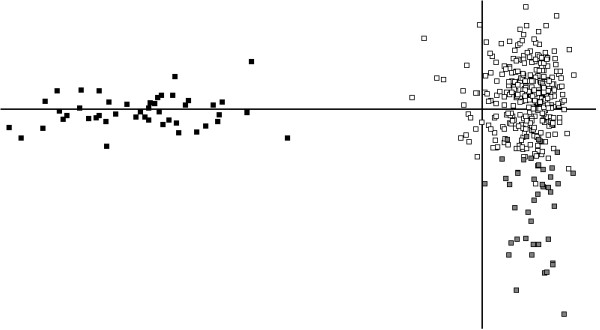
**Two-dimensional plot of a Factorial Correspondence Analysis based on allele differences at 8 microsatellites**. Horizontal axis: FC1 (60.4%); vertical axis: FC2 (11.5%) White squares: *An. atroparvus*; grey squares: *Anopheles atroparvus *from Romania; black squares: *An. maculipennis *s.s.

Comparisons involving *An. maculipennis *s.s. were all significant and gave *F*_*ST *_estimates above 0.200, *i.e. *more than two-fold greater than those among *An. atroparvus *samples. This differentiation was evident in the FCA (Figure 3) where *An. maculipennis *s.s. individuals form a cluster clearly separated from *An. atroparvus*. For the later species there was a nearly complete overlapping between samples, with the exception of the sample from Romania. Individuals from this sample tended to group together in a cluster that only partially overlapped with the remaining *An. atroparvus *samples.

### Geometric morphometrics

**Figure 4:**
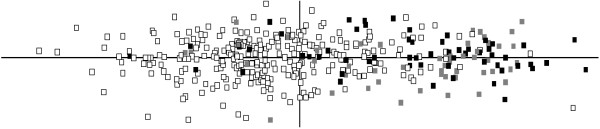
**Principal Component Analysis of tangent space coordinates derived from GPA of 21 wing landmarks**. Horizontal axis: PC1 (19.5%); vertical axis PC2 (15.13%). White squares: *An. atroparvus*; grey squares: *Anopheles atroparvus *from France; black squares: *An. maculipennis *s.s.

**Figure 5:**
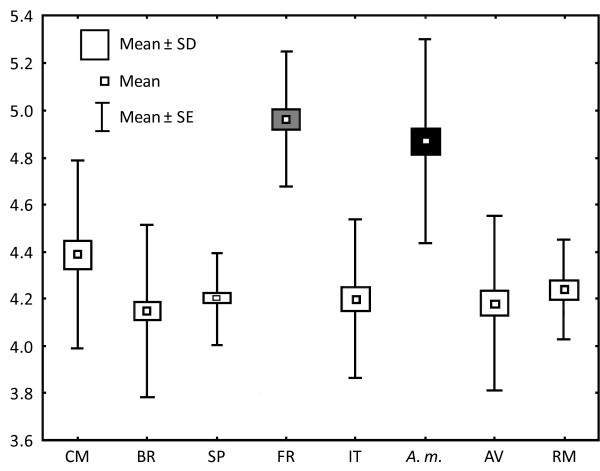
**Mean, standard deviation and error of centroid wing sizes**. CM: Castro Marim (portugal), BR: Barrancos (Portugal), SP: Tarragona (Spain), FR: Mèjanes (France), IT: Venice (Italy), *A. m.*: *An. maculipennis *s.s., AV: Aveiro (Portugal), RM: Salcioara (Romania). Grey box: *Anopheles atroparvus *from France; black box: *An. maculipennis *s.s.

When a PCA was conducted on the 21 wing landmarks, the two first PCs summarized 19.45% and 15.13% of the total variance, respectively. The first PC suggests some differences in the relative positions of the landmarks regarding the base of the wing. Main deformations centred on the medial of the wing on landmarks 13-14 and 15-16 (Figure 2). The distribution of individuals along the two first PCs is shown in Figure 4. There was major overlapping among individuals from all *An. atroparvus *samples. However, specimens of *An. maculipennis *s.s. and *An. atroparvus *from France tended to cluster more together along the positive axis of PC1 suggesting a higher phenotypic distance relative to the remaining *An. atroparvus *samples. The greater proximity between *An. maculipennis *s.s. and the French *An. atroparvus *sample agrees with the differences found in wing size between samples. Centroid sizes were used as measures of overall wing size differences among populations (Figure 5). The size differences among the populations were significant (Kruskal-Wallis Test: *H *= 176.6, *P *< 0.001) with the samples of France and *An. maculipennis *s.s. displaying considerably larger wings.

Pairwise phenotypic differentiation among *An. atroparvus *samples were quantified by estimates of the squared Mahalanobis distance (Table 1). Although the lowest and highest *D*_*M *_values matched with comparisons between the closest (Castro Marim *vs. *Barrancos; 108 km) and farthest samples (Castro Marim *vs. *Romania; 3,136 km), respectively, there was no clear pattern between phenotypic differentiation and the origin of the samples. Accordingly, there was no significant correlation between *D*_*M *_estimates and geographic distance between collection sites (Mantel Test: *P *= 0.073). No significant correlation was also observed between Mahalanobis distances and genetic distances as measured by pairwise *F*_*ST *_(Mantel Test: *P *= 0.658). As opposed to genetic differentiation, comparisons between *An. maculipennis *s.s. and *An. atroparvus *did not produce the highest *D*_*M *_values.

## Discussion

Microsatellite analysis of eight European samples of *An. atroparvus *indicates levels of genetic diversity similar to those described for other anopheline species of tropical regions, particularly from sub-Saharan Africa. The estimates of mean expected heterozygosity (0.61≤ *H*_*e *_≤0.73) are within the range of those obtained for the Afrotropical primary malaria vectors *Anopheles gambiae *s.s. (0.57≤ *H*_*e *_≤0.71; [31]), *Anopheles arabiensis *(0.65 ≤ *H*_*e *_≤0.78; [32]) and *Anopheles funestus *(0.64≤ *H*_*e *_≤0.78; [33]). In temperate climates, anopheline populations display marked seasonal variations in abundance, reaching high densities only during the summer months [7,34]. The high levels of genetic diversity suggest that *An. atroparvus *populations are able to maintain large effective population sizes in spite of the marked seasonality imposed by the winter cold temperatures. A similar scenario is also met by Afrotropical vector populations in dry savanna/sahelian regions. In *An. arabiensis*, the strong seasonal fluctuations in abundance do not seem to affect the overall genetic diversity and current effective population size in dry areas of Sudan and Senegal, where rains last for less than five months [35,36].

Estimates of genetic differentiation among *An. atroparvus *samples spanning over 3,000 km suggest a shallow population structure weakly correlated with geographic distance. This was evident when the most distant sample of Romania was excluded from the isolation-by-distance analysis. In addition, there was no particular pattern of population subdivision that could be attributable to the presence of two potential barriers to gene flow, the Pyrenees and the Alps. These mountain chains physically isolate the populations from the Iberian and Italian Peninsulas, respectively. For example, *F*_*ST *_estimates between France and Portugal (0.010-0.016) were considerably lower than those between Portugal and Spain (0.048-0.056).

The shallow patterns of population structure here reported for *An. atroparvus *are consistent with those observed in most primary malaria vector species from tropical climates (reviewed in [37]). Among the possible reasons for these patterns are historical demographic perturbations, particularly population expansions. These events may mask current levels of population structure and gene flow by disrupting the balance between migration and drift [37]. Evidence for recent population expansions have been documented for several malaria vectors such as *An. gambiae *s.s. and *An. arabiensis *[38], *Anopheles dirus *A and D [39] and *Anopheles minimus *[40]. In the two later examples, the signatures of population expansion have been associated with Pleistocene climate changes. This scenario can also be hypothesized for *An. atroparvus *as these populations were most likely affected by the Last Glacial Maximum, *ca. *18,000 years ago. In addition, population perturbations could also derive from the vector control actions implemented by the European malaria eradication programmes of the 1950's. Therefore, it is possible that the observed patterns of differentiation reflect differences in demographic history rather than contemporary gene flow among populations.

In contrast with within species comparisons, microsatellites revealed high differentiation between *An. atroparvus *samples and the only *An. maculipennis *s.s. sample analysed, a result consistent with their sibling species status. This was particularly evident by the FCA analysis in which there was a complete cluster separation between the two species. Values of *F*_*ST *_between 0.20 and 0.30 are similar those described between other anopheline sibling species (*e.g. An. gambiae/An. arabiensis*: 0.25, [41]; *An. dirus *complex: 0.21-0.39, [42]).

Phenotypic differentiation between *An. atroparvus *and *An. maculipennis *s.s. was not so evident. There was a partial overlapping between the clusters of the two species in the PCA. Furthermore, most *An. atroparvus *specimens of France shared the same dimensional space with *An. maculipennis s.s*. With the exception of this comparison, all the remaining *An. atroparvus *samples had significantly lower wing centroid sizes. These results concur with the notion of a relatively recent divergence time among the Palearctic members of the *An. maculipennis *complex not sufficient for the accumulation of phenotypic differences, in contrast to that of the Nearctic members of the complex [43,44]. A similar pattern was also observed by multivariate morphometric analysis between the recently separated *An. gambiae *s.s. and *An. arabiensis *in which the later species displayed significantly larger measures but still with overlapping distributions [45].

Within *An. atroparvus*, low to moderate levels of phenotypic differentiation were detected between samples by pairwise estimates of *D*_*M*_. However, there was no correlation between phenotypic differentiation and geographic or genetic distances. In mosquito populations, phenotypic variation is influenced by an assortment of environmental factors that include temperature, altitude, nutritional factors at the immature stages and host population distribution [45-47]. The levels of phenotypic variation in our samples are more likely to reflect the local environmental pressures to which these populations are subjected.

## Conclusions

The genetic and phenotypic variation among populations of the former European malaria vector *An. atroparvus *were analysed for the first time over a range of more than 3,000 km.The low levels of differentiation observed were not correlated with geographic distance or with potential physical barriers separating these populations. While these results may suggest considerable levels of gene flow, other explanations such as the effect of historical population perturbations can be hypothesized. Further genetic studies involving the analysis of temporal samples of *An. atroparvus *will help clarifying the recent demographic history of this species. In addition, analysis should also be extended to northern European locations, where *An. atroparvus *populations are also established and sometimes display biological differences [7]. Such analysis would provide new insights on the effect of temperature clines in the genetic structure of this vector. This will be essential to more precisely determine the degree of contemporary gene flow and hence the potential for mosquito-mediated spread of malaria parasites in the event of a focal re-establishment of malaria transmission. Likewise, it remains to be ascertained which local factors are governing phenotypic variation among these populations and how these may impact mosquito physiological and bio-ecological traits influencing vectorial capacity.

## Competing interests

The authors declare that they have no competing interests.

## Authors' contributions

JLV, CAS, EF, JML, CT, HB, MDL, LT, RA, MDB, SM-C, DB, RR, GN and DF carried out mosquito surveys and morphological identification of *An. maculipennis *s.l. samples. Molecular analyses were done by JLV, CAS, RA, PS and TLS. JP, PS and JLV carried out the statistical analysis of the genetic data. Geometric morphometrics was performed by BA, SSC, BD and NO. The study was conceived and design by JP, BA, DF, VER, MDB, RR and GN. JP, JLV, PS, BA and BD drafted the manuscript with the contributions of TLS, VER and RA. All authors read and approved the manuscript.

## Supplementary Material

Additional file 1 **Estimates of microsatellite genetic variability of *Anopheles atroparvus *in Europe **ATROdiversity.pdf portable document file (*.pdf) Estimates of microsatellite genetic variability of *Anopheles atroparvus *in Europe Estimates of number of alleles, expected heterozigosity and inbreeding coefficient (*F*_*IS*_) for 8 microsatellites. Click here for file
