# Supplementary material for: Genetic and phenotypic variation of the malaria vector Anopheles atroparvus in southern Europe
Source: Malar J. 2011 Jan 11;10:5. doi: 10.1186/1475-2875-10-5 (PMC3025906; doi:10.1186/1475-2875-10-5)
Supplement: Additional file 1 — Estimates of microsatellite genetic variability of Anopheles atroparvus in Europe ATROdiversity.pdf portable document file (*.pdf) Estimates of microsatellite genetic variability of Anopheles atroparvus in Europe Estimates of number of alleles, expected heterozigosity and inbreeding coefficient (FIS) for 8 microsatellites. [file 1475-2875-10-5-S1.PDF]

Estimates of microsatellite genetic variability of *Anopheles atroparvus* in Europe

		**MacuI3**	**MacuQ72**	**MacuG66**	**MacuU182**	**MacuUF**	**MacuW161**	**MacuO177**	**MacuGQ**	**All Loci**
Castro Marim	*A*	13	4	5	14	4	6	6	6	7
	H_e	0.826	0.676	0.627	0.827	0.734	0.576	**0.751**	**0.658**	**0.709**
	F_{is}	0.085	0.046	0.239	0.140	-0.239	-0.157	0.381	0.460	0.122
Barrancos	*A*	13	4	5	8	4	6	6	6	7
	H_e	0.854	0.661	**0.577**	0.730	0.610	0.646	0.769	**0.644**	**0.686**
	F_{is}	0.167	0.159	0.436	0.012	-0.229	0.085	0.274	0.400	0.163
Comporta	*A*	14	8	5	13	4	4	8	6	8
	H_e	0.897	0.713	0.623	0.829	0.641	0.698	**0.845**	0.625	**0.734**
	F_{is}	0.182	0.178	0.287	-0.019	0.029	0.140	0.327	0.238	0.170
Aveiro	*A*	16	3	6	12	4	6	7	7	8
	H_e	0.854	0.590	**0.554**	0.792	0.639	0.681	**0.757**	0.692	**0.695**
	F_{is}	0.201	0.247	0.426	-0.011	0.235	-0.110	0.501	0.174	0.202
Tarragona	*A*	14	6	4	11	2	6	6	4	7
	H_e	0.840	0.653	0.473	0.854	0.280	0.645	0.702	0.560	0.626
	F_{is}	-0.006	0.183	0.060	-0.035	-0.189	0.173	0.177	-0.088	0.049
Mèjanes	*A*	12	4	4	11	4	8	7	7	7
	H_e	0.825	0.555	**0.590**	0.846	0.646	0.690	**0.810**	0.674	**0.705**
	F_{is}	0.084	0.166	0.473	-0.103	-0.372	-0.021	0.411	0.164	0.096
Venice	*A*	8	5	5	9	3	4	5	5	6
	H_e	0.793	**0.668**	0.657	0.833	0.483	0.524	**0.656**	**0.617**	**0.654**
	F_{is}	0.019	0.501	0.066	-0.014	-0.474	0.000	0.377	0.531	0.139
Salcioara	*A*	10	5	5	9	3	3	8	6	6
	H_e	0.820	**0.530**	0.565	0.826	0.239	0.383	0.756	**0.745**	**0.608**
	F_{is}	-0.129	0.161	0.764	-0.046	0.070	0.304	0.148	0.429	0.193
All *An. atroparvus* samples	*A*	23	12	8	21	11	11	10	10	13
	H_e	**0.876**	**0.655**	**0.617**	0.820	0.588	0.649	**0.800**	**0.682**	**0.711**
	F_{is}	0.118	0.199	0.323	0.013	-0.108	0.019	0.242	0.213	0.129
Montalegre (*An. maculipennis* s.s.)	*A*	7	7	2	5	11	3	3	4	5
	H_e	0.529	0.752	0.164	0.709	0.842	**0.458**	0.390	0.191	**0.504**
	F_{is}	0.075	0.172	-0.086	0.123	0.190	0.563	-0.083	0.169	0.164
P (R_{ST} > pR_{ST})		0.319	0.414	0.718	0.227	0.015	0.624	0.007	0.028	0.029

A: number of alleles. H_e: unbiased estimate of expected heterozygosity. Significant departures from H-W proportions after Bonferroni corrections are indicated in bold. F_{is}: Inbreeding coefficient. All Loci: mean values over loci. P (R_{ST} > pR_{ST}): probability of a one-sided permutation test to evaluate the influence of stepwise mutations in the differentiation among samples (see Methods).
